# 1-{[Dimeth­yl(phen­yl)sil­yl]meth­yl}-3-(2-phenyl­eth­yl)-1*H*-benzimidazol-3-ium bromide monohydrate

**DOI:** 10.1107/S1600536812034915

**Published:** 2012-08-15

**Authors:** Mehmet Akkurt, Hasan Küçükbay, Nihat Şireci, Orhan Büyükgüngör

**Affiliations:** a Department of Physics, Faculty of Sciences, Erciyes University, 38039 Kayseri, Turkey; b Department of Chemistry, Faculty of Arts and Sciences, Ínönü University, 44280 Malatya, Turkey; c Department of Chemistry, Faculty of Education, Adiyaman University, 02040 Adiyaman, Turkey; d Department of Physics, Faculty of Arts and Sciences, Ondokuz Mayıs University, 55139 Samsun, Turkey

## Abstract

The title compound, C_24_H_27_N_2_Si^+^·Br^−^·H_2_O, was synthesized from 1-(dimethyl­phenyl­silylmeth­yl)-1*H*-benzimidazole and (2-bromo­eth­yl)benzene in dimethyl­formamide. The benzimidazole ring system is nearly planar, with a maximum deviation of 0.015 (5) Å, and forms dihedral angles of 73.0 (3) and 39.6 (2)°, with the phenyl rings. In the crystal, mol­ecules are linked by O—H⋯Br, C—H⋯Br and C—H⋯O hydrogen bonds. In addition, the structure features π–π stacking inter­actions, with a face-to-face separation of 3.644 (3) Å between parallel benzimidazole ring systems.

## Related literature

For general background to benzimidazole derivatives, see: Lukevics *et al.* (2001 ▶); Tavman *et al.* (2005 ▶); Küçükbay *et al.* (1995 ▶, 2004 ▶, 2010 ▶, 2011 ▶); Yılmaz *et al.* (2011 ▶); Çetinkaya *et al.* (1996 ▶). For similar structures, see: Akkurt *et al.* (2010*a* ▶,*b* ▶); Baktır *et al.* (2010 ▶).
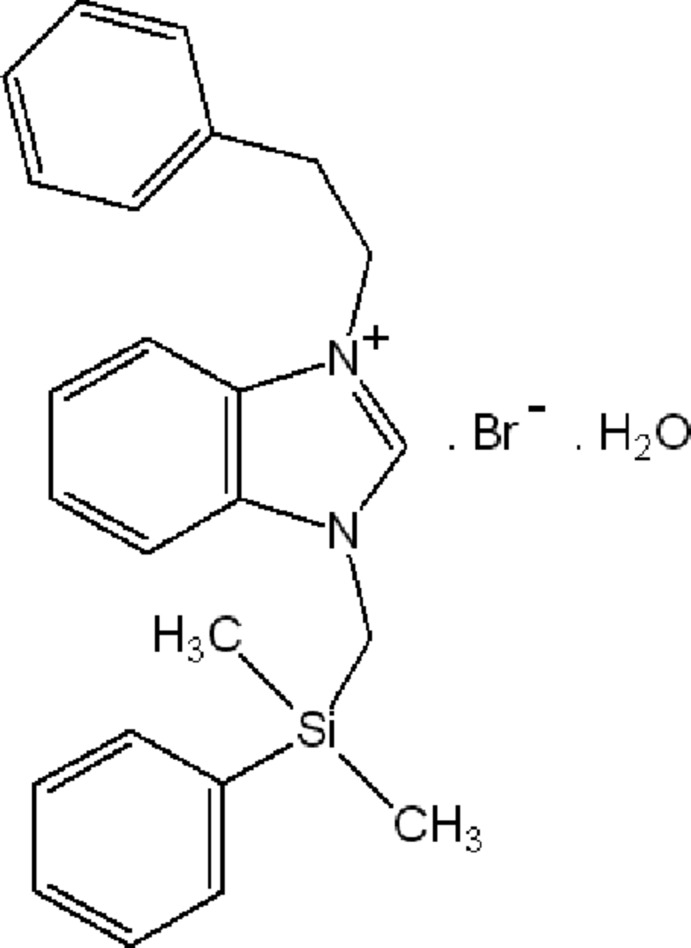


## Experimental

### 

#### Crystal data

C_24_H_27_N_2_Si^+^·Br^−^·H_2_O
*M* *_r_* = 469.48
Monoclinic, 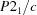

*a* = 15.1750 (11) Å
*b* = 8.9097 (6) Å
*c* = 17.9440 (14) Å
β = 96.235 (6)°
*V* = 2411.8 (3) Å^3^
*Z* = 4
Mo *K*α radiation
μ = 1.77 mm^−1^
*T* = 296 K
0.26 × 0.20 × 0.13 mm

#### Data collection

Stoe IPDS 2 diffractometer
Absorption correction: integration (*X-RED32*; Stoe & Cie, 2002 ▶) *T*_min_ = 0.656, *T*_max_ = 0.803
19519 measured reflections
5536 independent reflections
3023 reflections with *I* > 2σ(*I*)
*R*_int_ = 0.074

#### Refinement

*R*[*F* ^2^ > 2σ(*F* ^2^)] = 0.069
*wR*(*F* ^2^) = 0.173
*S* = 1.03
5536 reflections
244 parameters
3 restraints
H atoms treated by a mixture of independent and constrained refinement
Δρ_max_ = 0.82 e Å^−3^
Δρ_min_ = −0.53 e Å^−3^

### 

Data collection: *X-AREA* (Stoe & Cie, 2002 ▶); cell refinement: *X-AREA*; data reduction: *X-RED32* (Stoe & Cie, 2002 ▶); program(s) used to solve structure: *SIR97* (Altomare *et al.*, 1999 ▶); program(s) used to refine structure: *SHELXL97* (Sheldrick, 2008 ▶); molecular graphics: *ORTEP-3* (Farrugia, 1997 ▶); software used to prepare material for publication: *WinGX* (Farrugia, 1999 ▶).

## Supplementary Material

Crystal structure: contains datablock(s) global, I. DOI: 10.1107/S1600536812034915/nr2031sup1.cif

Structure factors: contains datablock(s) I. DOI: 10.1107/S1600536812034915/nr2031Isup2.hkl

Supplementary material file. DOI: 10.1107/S1600536812034915/nr2031Isup3.cml

Additional supplementary materials:  crystallographic information; 3D view; checkCIF report

## Figures and Tables

**Table 1:** Hydrogen-bond geometry (Å, °)

*D*—H⋯*A*	*D*—H	H⋯*A*	*D*⋯*A*	*D*—H⋯*A*
O*W*1—H*W*1⋯Br1	0.83 (6)	2.57 (7)	3.365 (5)	160 (6)
O*W*1—H*W*2⋯Br1^i^	0.83 (6)	2.56 (7)	3.384 (5)	175 (6)
C7—H7⋯Br1	0.93	2.85	3.597 (5)	138
C8—H8*A*⋯Br1^ii^	0.97	2.89	3.822 (5)	160
C16—H16*B*⋯O*W*1^iii^	0.97	2.55	3.494 (7)	164

Symmetry codes: (i) 
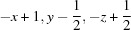
; (ii) 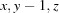; (iii) 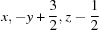.

**Fig. 1.**
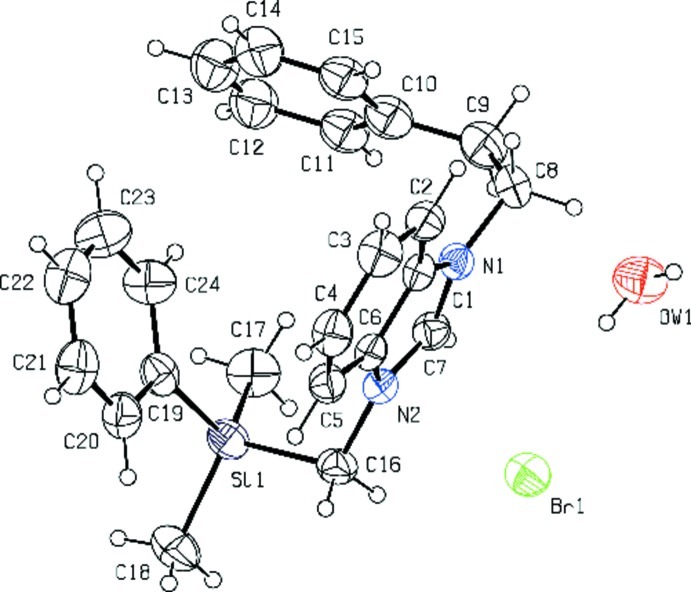
The molecule of the title compound (I), showing the atom labelling scheme. Displacement ellipsoids for non-H atoms are drawn at the 30% probability level.

**Fig. 2.**
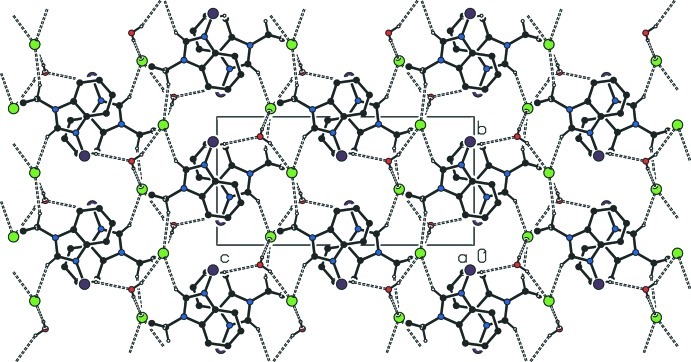
View of the packing and hydrogen bonding interactions of (I) along the a axis in the unitcell. All hydrogen atoms not involved in hydrogen bonding, two phenyl rings and two ethyl groups have been omitted for clarity.

**Fig. 3.**
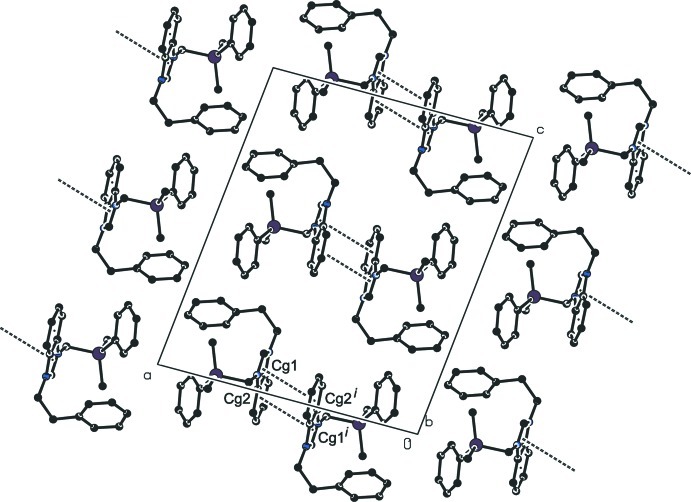
A view of the intermolecular face-to-face π–π stacking interactions between parallel benzimidazole ring systems, along the b axis in the crystal structure of (I). All hydrogen atoms, bromide ions and water molecules has been omitted for clarity. [Cg1 and Cg2 are the centroids of the N1/N2/C1/C6/C7 and C1–C6 rings, respectively. Symmetry code: (i) = 1 - x, 1 - y, -z].

## supplementary crystallographic information

### Comment

Heterocyclic compounds play an important role in biological systems and in many industrial fields. One of the these type heterocyclic compounds, benzimidazole is also an important pharmacophore in new drug designed and synthesized (Tavman *et al.*, 2005; Küçükbay *et al.*, 2004). Benzimidazol containing compounds o display a wide range of pharmacological activities, and are used for therapeutic purposes as anti-fungal, anti-bacterial, anti-helmintic, hypotensive, vasodilator, spasmolytic and anti-ulser activities. We also investigated their some anti-microbial activities (Küçükbay *et al.*, 1995; Çetinkaya *et al.*, 1996; Küçükbay *et al.*, 2010; Küçükbay *et al.*, 2011; Yılmaz *et al.*, 2011). Alkylsilyl substituted benzimidazole derivatives exhibit important *in vitro* cytotoxic activity. For example, 1-(3-trimethylsilylpropyl)benzimidazole inhibits carcinoma S180 tumour growth in dose 1 mg.kg^-1^ by 62% (on ICR mice) (Lukevics *et al.*, 2001). The objective of this study is to synthesize and to elucidate the crystal structure of a new silyl benzimidazole compound.

In the title compound (I), (Fig. 1), the benzimidazole ring system (N1/N2/C1–C7) is nearly planar with a maximum deviation of 0.015 (5) Å for C6. The dihedral angle between the two terminal phenyl rings (C10–C15 and C19–C24) is 77.1 (3)°. The orientation of the two phenyl rings towards the benzimidazole-ring is remarkable [73.0 (3)° and 39.6 (2)°, respectively]. The values of the bond lengths and bond angles in (I) are in normal range, and they are in a good agreement with those found in similar compounds (Akkurt *et al.*, 2010*a*,*b*; Baktır *et al.*, 2010). The Si atom adopts distorted tetrahedral geometries in (I) and the angles around the Si atoms vary from 108.4 (3)° to 112.12 (17)°.

In the crystal, molecules are linked by O···H···Br, C—H···Br and C—H···O hydrogen bonds (Table 1 and Fig. 2). Furthermore, face-to-face π-π stacking interactions between parallel benzimidazole ring systems [Fig.3; *Cg*1···*Cg*2(1 - *x*, 1 - *y*, -*z*) = 3.644 (3) Å; where *Cg*1 and *Cg*2 are the centroids of the N1/N2/C1/C6/C7 and C1–C6 rings, respectively] help to the stabilization of the crystal structure.

### Experimental

A mixture of 1-(dimethylphenylsilylmethyl)benzimidazole (1.34 g, 5.0 mmol) and (2-bromoethyl)benzene (0.7 ml, 5.1 mmol) in dimethylformamide (5 ml) was refluxed for 3 h. The mixture was then cooled and the volatiles were removed under vacuum. The residue was crystallized from a dimethylformamide/ethanol (1:1). White crystals of the title compound (2.05 g, 87%) were obtained, mp 415–416 K: υ_(C=N)_ = 1557 cm^-1^. Anal. found: C 61.12, H 6.21, N 5.56%. Calculated for C_24_H_29_BrN_2_OSi: C 61.40, H 6.23, N 5.97%. ^1^H NMR (δ, DMSO-d_6_): 9.51 (s, 1H, NCHN), 8.02–7.99 (m, 2H, C_6_H_4_ ), 7.84–7.81 (m, 2H, C_6_H_4_ ), 7.62–7.19 (m, 10H, C_6_H_5_ phenethyl and 5H C_6_H_5_Si), 4.78 (t, 2H, CH_2_ phenethyl, *J*= 7.2 Hz), 4.38 (s, 2H, CH_2_Si), 3,20 (t, 2H, CH_2_ phenethyl, *J*= 7.2 Hz) and 0.31 (s, 6H, Si(CH_3_)_2_). ^13^C NMR (δ, DMSO-d_6_): 141.3 (NCHN), 137.3, 134.7, 134.3, 131.9, 131.1, 130.5, 129.2, 129.1, 128.5, 127.4, 126.9, 126.5, 114.3 and 114.0 (C_6_H_4_—ArH; C_6_H_5_– phenethyl and C_6_H_5_Si), 47.9 (CH_2_ phenethyl), 37.9 (CH_2_Si) 35.1 (CH_2_ phenethyl), and -3.9 (Si(CH_3_)_2_).

### Refinement

The water H atoms were located from a difference Fourier map and refined with distance restraints of O—H = 0.83 (2) Å and H···H = 1.35 (2) Å, and with *U*_iso_(H) = 1.5 *U*_eq_(O). The H atoms bonded to carbon atoms were positioned geometrically, with C—H = 0.93 Å (aromatic), 0.96 Å (methyl) and 0.97 Å (methylene) H atoms, respectively, and refined as riding with *U*_iso_(H) = 1.5 *U*_eq_(C) for the methyl groups and *U*_iso_(H) = 1.2 *U*_eq_(C) for others groups. Nine poorly fitted reflections (-2 0 2, -2 1 1, 7 2 0, 3 2 1, 0 1 6, 16 0 0, -4 0 2, -2 4 4, -2 1 7) were omitted from the refinement.

### Crystal data

**Table:**

C_24_H_27_N_2_Si^+^·Br^−^·H_2_O	*F*(000) = 976
*M**_r_* = 469.48	*D*_x_ = 1.293 Mg m^−^^3^
Monoclinic, *P*2_1_/*c*	Mo *K*α radiation, λ = 0.71073 Å
Hall symbol: -P 2ybc	Cell parameters from 16623 reflections
*a* = 15.1750 (11) Å	θ = 1.9–28.0°
*b* = 8.9097 (6) Å	µ = 1.77 mm^−^^1^
*c* = 17.9440 (14) Å	*T* = 296 K
β = 96.235 (6)°	Prism, colourless
*V* = 2411.8 (3) Å^3^	0.26 × 0.20 × 0.13 mm
*Z* = 4	

### Data collection

**Table:**

Stoe IPDS 2 diffractometer	5536 independent reflections
Radiation source: sealed X-ray tube, 12 x 0.4 mm long-fine focus	3023 reflections with *I* > 2σ(*I*)
Plane graphite monochromator	*R*_int_ = 0.074
Detector resolution: 6.67 pixels mm^-1^	θ_max_ = 27.7°, θ_min_ = 2.3°
ω scans	*h* = −19→19
Absorption correction: integration (*X-RED32*; Stoe & Cie, 2002)	*k* = −11→11
*T*_min_ = 0.656, *T*_max_ = 0.803	*l* = −23→22
19519 measured reflections	

### Refinement

**Table:**

Refinement on *F*^2^	Primary atom site location: structure-invariant direct methods
Least-squares matrix: full	Secondary atom site location: difference Fourier map
*R*[*F*^2^ > 2σ(*F*^2^)] = 0.069	Hydrogen site location: inferred from neighbouring sites
*wR*(*F*^2^) = 0.173	H atoms treated by a mixture of independent and constrained refinement
*S* = 1.03	*w* = 1/[σ^2^(*F*_o_^2^) + (0.0795*P*)^2^ + 0.0123*P*] where *P* = (*F*_o_^2^ + 2*F*_c_^2^)/3
5536 reflections	(Δ/σ)_max_ < 0.001
244 parameters	Δρ_max_ = 0.82 e Å^−^^3^
3 restraints	Δρ_min_ = −0.53 e Å^−^^3^

### Special details

**Table:**

Geometry. Bond distances, angles *etc*. have been calculated using the rounded fractional coordinates. All su's are estimated from the variances of the (full) variance-covariance matrix. The cell e.s.d.'s are taken into account in the estimation of distances, angles and torsion angles
Refinement. Refinement on *F*^2^ for ALL reflections except those flagged by the user for potential systematic errors. Weighted *R*-factors *wR* and all goodnesses of fit *S* are based on *F*^2^, conventional *R*-factors *R* are based on *F*, with *F* set to zero for negative *F*^2^. The observed criterion of *F*^2^ > σ(*F*^2^) is used only for calculating -*R*-factor-obs *etc*. and is not relevant to the choice of reflections for refinement. *R*-factors based on *F*^2^ are statistically about twice as large as those based on *F*, and *R*-factors based on ALL data will be even larger.

### Fractional atomic coordinates and isotropic or equivalent isotropic displacement parameters (Å^2^)

**Table:**

	*x*	*y*	*z*	*U*_iso_*/*U*_eq_	
Si1	0.78140 (10)	0.80539 (15)	0.01520 (8)	0.0622 (5)	
N1	0.6284 (2)	0.4424 (4)	0.1286 (2)	0.0494 (11)	
N2	0.6390 (2)	0.6253 (4)	0.0489 (2)	0.0504 (11)	
C1	0.6113 (3)	0.3800 (5)	0.0576 (3)	0.0485 (14)	
C2	0.5887 (3)	0.2349 (5)	0.0337 (3)	0.0615 (19)	
C3	0.5739 (4)	0.2140 (6)	−0.0423 (3)	0.071 (2)	
C4	0.5800 (4)	0.3300 (6)	−0.0928 (3)	0.0676 (19)	
C5	0.6016 (3)	0.4728 (6)	−0.0695 (3)	0.0598 (17)	
C6	0.6175 (3)	0.4968 (5)	0.0065 (3)	0.0479 (14)	
C7	0.6444 (3)	0.5879 (5)	0.1201 (3)	0.0550 (17)	
C8	0.6288 (3)	0.3604 (6)	0.1995 (3)	0.0611 (17)	
C9	0.7112 (3)	0.3908 (4)	0.2537 (2)	0.092 (3)	
C10	0.7978 (3)	0.3669 (4)	0.2238 (2)	0.0805 (14)	
C11	0.8658 (3)	0.4673 (4)	0.2459 (2)	0.0805 (14)	
C12	0.9508 (5)	0.4492 (9)	0.2211 (5)	0.103 (3)	
C13	0.9606 (5)	0.3318 (9)	0.1751 (5)	0.104 (3)	
C14	0.8930 (5)	0.2323 (9)	0.1538 (5)	0.105 (3)	
C15	0.8145 (5)	0.2532 (7)	0.1779 (3)	0.0805 (14)	
C16	0.6585 (3)	0.7756 (5)	0.0205 (3)	0.0600 (16)	
C17	0.8383 (5)	0.8082 (7)	0.1119 (3)	0.088 (3)	
C18	0.7921 (2)	0.9879 (4)	−0.03326 (19)	0.090 (3)	
C19	0.8198 (2)	0.6457 (4)	−0.03916 (19)	0.0629 (17)	
C20	0.7951 (2)	0.6338 (4)	−0.11654 (19)	0.073 (2)	
C21	0.8174 (4)	0.5106 (8)	−0.1566 (4)	0.087 (3)	
C22	0.8647 (5)	0.3947 (8)	−0.1216 (4)	0.093 (3)	
C23	0.8905 (5)	0.4028 (7)	−0.0465 (5)	0.102 (3)	
C24	0.8673 (4)	0.5258 (7)	−0.0051 (4)	0.085 (3)	
Br1	0.59570 (4)	0.93585 (6)	0.20869 (3)	0.0720 (2)	
OW1	0.5856 (3)	0.6540 (5)	0.3323 (3)	0.0960 (19)	
H2	0.58390	0.15670	0.06740	0.0740*	
H3	0.55930	0.11850	−0.06060	0.0850*	
H4	0.56900	0.31030	−0.14380	0.0810*	
H5	0.60550	0.55040	−0.10370	0.0720*	
H7	0.65760	0.65480	0.15950	0.0660*	
H8A	0.62500	0.25360	0.18900	0.0740*	
H8B	0.57670	0.38860	0.22320	0.0740*	
H9A	0.70880	0.49390	0.27070	0.1100*	
H9B	0.70910	0.32680	0.29720	0.1100*	
H11	0.85550	0.54710	0.27720	0.0970*	
H12	0.99720	0.51450	0.23580	0.1230*	
H13	1.01510	0.31750	0.15710	0.1240*	
H14	0.90240	0.15150	0.12280	0.1260*	
H15	0.76900	0.18660	0.16250	0.0970*	
H16A	0.63720	0.85100	0.05320	0.0720*	
H16B	0.62690	0.78870	−0.02900	0.0720*	
H17A	0.81720	0.89180	0.13870	0.1320*	
H17B	0.90100	0.81790	0.11010	0.1320*	
H17C	0.82610	0.71650	0.13690	0.1320*	
H18A	0.77150	1.06730	−0.00340	0.1350*	
H18B	0.75710	0.98570	−0.08120	0.1350*	
H18C	0.85310	1.00490	−0.04030	0.1350*	
H20	0.76290	0.71120	−0.14130	0.0870*	
H21	0.80020	0.50590	−0.20790	0.1050*	
H22	0.87920	0.31120	−0.14890	0.1110*	
H23	0.92380	0.32550	−0.02280	0.1230*	
H24	0.88370	0.52800	0.04640	0.1030*	
HW1	0.589 (5)	0.705 (8)	0.294 (3)	0.1450*	
HW2	0.542 (4)	0.598 (8)	0.325 (4)	0.1450*	

### Atomic displacement parameters (Å^2^)

**Table:**

	*U*^11^	*U*^22^	*U*^33^	*U*^12^	*U*^13^	*U*^23^
Si1	0.0692 (9)	0.0560 (7)	0.0610 (9)	−0.0105 (7)	0.0047 (7)	0.0011 (6)
N1	0.052 (2)	0.0482 (18)	0.049 (2)	0.0002 (17)	0.0098 (16)	−0.0020 (17)
N2	0.051 (2)	0.0471 (18)	0.054 (2)	0.0046 (16)	0.0102 (18)	0.0005 (17)
C1	0.038 (2)	0.055 (2)	0.053 (3)	0.0007 (18)	0.007 (2)	−0.005 (2)
C2	0.058 (3)	0.053 (3)	0.074 (4)	−0.003 (2)	0.010 (3)	−0.004 (2)
C3	0.074 (4)	0.064 (3)	0.074 (4)	−0.009 (3)	0.004 (3)	−0.021 (3)
C4	0.067 (3)	0.082 (4)	0.054 (3)	−0.003 (3)	0.007 (3)	−0.015 (3)
C5	0.056 (3)	0.069 (3)	0.055 (3)	0.002 (2)	0.009 (2)	0.004 (2)
C6	0.037 (2)	0.055 (2)	0.052 (3)	0.0032 (19)	0.0064 (19)	−0.003 (2)
C7	0.059 (3)	0.048 (3)	0.059 (3)	0.001 (2)	0.011 (2)	−0.007 (2)
C8	0.064 (3)	0.067 (3)	0.054 (3)	−0.010 (2)	0.014 (2)	0.001 (2)
C9	0.100 (5)	0.103 (5)	0.073 (4)	0.004 (4)	0.014 (4)	0.014 (3)
C10	0.088 (3)	0.081 (2)	0.070 (2)	−0.0050 (18)	−0.0026 (19)	0.0080 (16)
C11	0.088 (3)	0.081 (2)	0.070 (2)	−0.0050 (18)	−0.0026 (19)	0.0080 (16)
C12	0.074 (5)	0.111 (6)	0.122 (6)	−0.013 (4)	0.005 (4)	0.024 (5)
C13	0.070 (4)	0.119 (6)	0.120 (6)	−0.014 (4)	0.003 (4)	0.017 (5)
C14	0.095 (5)	0.107 (5)	0.116 (6)	−0.003 (4)	0.020 (4)	0.015 (4)
C15	0.088 (3)	0.081 (2)	0.070 (2)	−0.0050 (18)	−0.0026 (19)	0.0080 (16)
C16	0.074 (3)	0.046 (2)	0.061 (3)	0.004 (2)	0.012 (3)	0.002 (2)
C17	0.098 (5)	0.087 (4)	0.076 (4)	−0.004 (3)	−0.010 (3)	−0.011 (3)
C18	0.112 (5)	0.068 (3)	0.092 (5)	−0.018 (3)	0.019 (4)	0.015 (3)
C19	0.054 (3)	0.069 (3)	0.065 (3)	−0.017 (2)	0.004 (2)	0.002 (3)
C20	0.062 (4)	0.086 (4)	0.071 (4)	−0.004 (3)	0.012 (3)	0.000 (3)
C21	0.072 (4)	0.114 (5)	0.077 (4)	−0.008 (4)	0.012 (3)	−0.018 (4)
C22	0.081 (5)	0.093 (4)	0.105 (6)	−0.011 (4)	0.016 (4)	−0.031 (4)
C23	0.110 (6)	0.080 (4)	0.114 (6)	0.012 (4)	−0.001 (5)	−0.010 (4)
C24	0.101 (5)	0.076 (4)	0.075 (4)	0.004 (3)	−0.007 (3)	−0.005 (3)
Br1	0.0904 (4)	0.0581 (3)	0.0677 (4)	−0.0022 (3)	0.0101 (3)	−0.0092 (3)
OW1	0.100 (4)	0.092 (3)	0.092 (3)	−0.004 (2)	−0.008 (3)	0.005 (2)

### Geometric parameters (Å, º)

**Table:**

Si1—C16	1.896 (5)	C22—C23	1.364 (11)
Si1—C17	1.853 (6)	C23—C24	1.391 (10)
Si1—C18	1.859 (4)	C2—H2	0.9300
Si1—C19	1.854 (4)	C3—H3	0.9300
OW1—HW1	0.83 (6)	C4—H4	0.9300
OW1—HW2	0.83 (6)	C5—H5	0.9300
N1—C8	1.467 (6)	C7—H7	0.9300
N1—C1	1.388 (6)	C8—H8B	0.9700
N1—C7	1.331 (6)	C8—H8A	0.9700
N2—C6	1.394 (6)	C9—H9B	0.9700
N2—C7	1.314 (6)	C9—H9A	0.9700
N2—C16	1.474 (6)	C11—H11	0.9300
C1—C2	1.393 (6)	C12—H12	0.9300
C1—C6	1.397 (7)	C13—H13	0.9300
C2—C3	1.371 (8)	C14—H14	0.9300
C3—C4	1.384 (8)	C15—H15	0.9300
C4—C5	1.368 (8)	C16—H16B	0.9700
C5—C6	1.376 (8)	C16—H16A	0.9700
C8—C9	1.523 (6)	C17—H17A	0.9600
C9—C10	1.488 (6)	C17—H17B	0.9600
C10—C11	1.391 (6)	C17—H17C	0.9600
C10—C15	1.347 (7)	C18—H18A	0.9600
C11—C12	1.419 (9)	C18—H18C	0.9600
C12—C13	1.351 (12)	C18—H18B	0.9600
C13—C14	1.378 (11)	C20—H20	0.9300
C14—C15	1.324 (11)	C21—H21	0.9300
C19—C24	1.392 (7)	C22—H22	0.9300
C19—C20	1.402 (5)	C23—H23	0.9300
C20—C21	1.374 (8)	C24—H24	0.9300
C21—C22	1.370 (10)		
			
C16—Si1—C17	108.4 (3)	C6—C5—H5	121.00
C16—Si1—C18	106.39 (18)	N2—C7—H7	124.00
C16—Si1—C19	106.61 (19)	N1—C7—H7	124.00
C17—Si1—C18	111.8 (2)	N1—C8—H8A	109.00
C17—Si1—C19	111.2 (2)	C9—C8—H8A	109.00
C18—Si1—C19	112.12 (17)	C9—C8—H8B	109.00
HW1—OW1—HW2	109 (7)	H8A—C8—H8B	108.00
C1—N1—C8	125.4 (4)	N1—C8—H8B	109.00
C7—N1—C8	127.0 (4)	C8—C9—H9A	108.00
C1—N1—C7	107.7 (4)	C10—C9—H9A	108.00
C6—N2—C7	108.0 (4)	C10—C9—H9B	108.00
C6—N2—C16	127.0 (4)	C8—C9—H9B	108.00
C7—N2—C16	124.9 (4)	H9A—C9—H9B	107.00
N1—C1—C2	132.0 (5)	C12—C11—H11	120.00
N1—C1—C6	106.6 (4)	C10—C11—H11	120.00
C2—C1—C6	121.4 (5)	C11—C12—H12	122.00
C1—C2—C3	116.1 (5)	C13—C12—H12	122.00
C2—C3—C4	122.3 (5)	C12—C13—H13	119.00
C3—C4—C5	121.7 (5)	C14—C13—H13	119.00
C4—C5—C6	117.3 (5)	C15—C14—H14	120.00
N2—C6—C1	106.4 (4)	C13—C14—H14	120.00
N2—C6—C5	132.4 (4)	C14—C15—H15	119.00
C1—C6—C5	121.2 (4)	C10—C15—H15	119.00
N1—C7—N2	111.4 (4)	Si1—C16—H16A	109.00
N1—C8—C9	113.0 (4)	Si1—C16—H16B	109.00
C8—C9—C10	116.1 (3)	N2—C16—H16B	109.00
C9—C10—C11	117.5 (3)	H16A—C16—H16B	108.00
C11—C10—C15	118.4 (5)	N2—C16—H16A	109.00
C9—C10—C15	124.1 (5)	Si1—C17—H17B	109.00
C10—C11—C12	120.6 (5)	Si1—C17—H17C	109.00
C11—C12—C13	116.4 (7)	H17A—C17—H17B	109.00
C12—C13—C14	122.7 (7)	H17A—C17—H17C	110.00
C13—C14—C15	119.2 (7)	H17B—C17—H17C	110.00
C10—C15—C14	122.8 (7)	Si1—C17—H17A	109.00
Si1—C16—N2	112.3 (3)	Si1—C18—H18A	109.00
Si1—C19—C20	120.9 (3)	Si1—C18—H18C	110.00
Si1—C19—C24	122.4 (4)	H18A—C18—H18B	109.00
C20—C19—C24	116.5 (4)	H18A—C18—H18C	110.00
C19—C20—C21	121.6 (4)	H18B—C18—H18C	109.00
C20—C21—C22	120.6 (6)	Si1—C18—H18B	109.00
C21—C22—C23	119.5 (7)	C21—C20—H20	119.00
C22—C23—C24	120.5 (7)	C19—C20—H20	119.00
C19—C24—C23	121.3 (6)	C20—C21—H21	120.00
C1—C2—H2	122.00	C22—C21—H21	120.00
C3—C2—H2	122.00	C23—C22—H22	120.00
C2—C3—H3	119.00	C21—C22—H22	120.00
C4—C3—H3	119.00	C22—C23—H23	120.00
C5—C4—H4	119.00	C24—C23—H23	120.00
C3—C4—H4	119.00	C19—C24—H24	119.00
C4—C5—H5	121.00	C23—C24—H24	119.00
			
C18—Si1—C19—C24	140.9 (4)	C2—C1—C6—C5	−0.1 (7)
C17—Si1—C19—C20	−170.9 (3)	C2—C1—C6—N2	178.6 (4)
C17—Si1—C16—N2	−67.2 (4)	N1—C1—C6—C5	−178.2 (4)
C16—Si1—C19—C20	71.1 (3)	C1—C2—C3—C4	−0.7 (8)
C19—Si1—C16—N2	52.7 (4)	C2—C3—C4—C5	0.2 (9)
C17—Si1—C19—C24	15.0 (5)	C3—C4—C5—C6	0.3 (8)
C18—Si1—C16—N2	172.5 (3)	C4—C5—C6—N2	−178.7 (5)
C16—Si1—C19—C24	−103.0 (4)	C4—C5—C6—C1	−0.3 (7)
C18—Si1—C19—C20	−44.9 (3)	N1—C8—C9—C10	−53.3 (5)
C7—N1—C1—C6	−0.4 (5)	C8—C9—C10—C11	141.8 (4)
C8—N1—C1—C2	2.1 (7)	C8—C9—C10—C15	−39.3 (6)
C1—N1—C8—C9	130.7 (4)	C11—C10—C15—C14	0.8 (8)
C7—N1—C8—C9	−49.0 (6)	C9—C10—C11—C12	178.4 (5)
C8—N1—C1—C6	179.9 (4)	C15—C10—C11—C12	−0.6 (7)
C1—N1—C7—N2	0.0 (5)	C9—C10—C15—C14	−178.1 (6)
C7—N1—C1—C2	−178.2 (5)	C10—C11—C12—C13	0.7 (10)
C8—N1—C7—N2	179.8 (4)	C11—C12—C13—C14	−1.1 (12)
C16—N2—C6—C1	175.7 (4)	C12—C13—C14—C15	1.3 (13)
C16—N2—C6—C5	−5.8 (8)	C13—C14—C15—C10	−1.2 (11)
C16—N2—C7—N1	−176.0 (4)	Si1—C19—C20—C21	−175.0 (4)
C7—N2—C6—C5	178.0 (5)	C24—C19—C20—C21	−0.5 (6)
C6—N2—C7—N1	0.3 (5)	Si1—C19—C24—C23	175.9 (5)
C7—N2—C6—C1	−0.5 (5)	C20—C19—C24—C23	1.5 (8)
C6—N2—C16—Si1	−92.5 (5)	C19—C20—C21—C22	0.2 (8)
C7—N2—C16—Si1	83.1 (5)	C20—C21—C22—C23	−0.7 (10)
N1—C1—C2—C3	178.1 (5)	C21—C22—C23—C24	1.6 (11)
C6—C1—C2—C3	0.6 (7)	C22—C23—C24—C19	−2.0 (10)
N1—C1—C6—N2	0.5 (5)		

### Hydrogen-bond geometry (Å, º)

**Table:**

*D*—H···*A*	*D*—H	H···*A*	*D*···*A*	*D*—H···*A*
O*W*1—H*W*1···Br1	0.83 (6)	2.57 (7)	3.365 (5)	160 (6)
O*W*1—H*W*2···Br1^i^	0.83 (6)	2.56 (7)	3.384 (5)	175 (6)
C7—H7···Br1	0.93	2.85	3.597 (5)	138
C8—H8*A*···Br1^ii^	0.97	2.89	3.822 (5)	160
C16—H16*B*···O*W*1^iii^	0.97	2.55	3.494 (7)	164

Symmetry codes: (i) −*x*+1, *y*−1/2, −*z*+1/2; (ii) *x*, *y*−1, *z*; (iii) *x*, −*y*+3/2, *z*−1/2.
